# Hepatitis C Virus Induced a Novel Apoptosis-Like Death of Pancreatic Beta Cells through a Caspase 3-Dependent Pathway

**DOI:** 10.1371/journal.pone.0038522

**Published:** 2012-06-04

**Authors:** Qian Wang, Jizheng Chen, Yun Wang, Xiao Han, Xinwen Chen

**Affiliations:** 1 State Key Lab of Virology, Wuhan Institute of Virology, Chinese Academy of Sciences, Wuhan, China; 2 Key Laboratory of Human Functional Genomics of Jiangsu Province, School of Basic Medical Science, Nanjing Medical University, Nanjing, China; Duke University School of Medicine, United States of America

## Abstract

Epidemiological and experimental studies have suggested that Hepatitis C virus (HCV) infection is associated with the development of type 2 diabetes. Pancreatic beta cell failure is central to the progression of type 2 diabetes. Using virus infection system, we investigate the influence of HCV infection on the fate of the insulinoma cell line, MIN6. Our experiments demonstrate that the HCV virion itself is indispensable and has a dose- and time-dependent cytopathic effect on the cells. HCV infection inhibits cell proliferation and induces death of MIN6 cells with apoptotic characteristics, including cell surface exposure of phosphatidylserine, decreased mitochondrial membrane potential, activation of caspase 3 and poly (ADP-ribose) polymerase, and DNA fragmentation in the nucleus. However, the fact that HCV-infected cells exhibit a dilated, low-density nucleus with intact plasma and nuclear membrane indicates that a novel apoptosis-like death occurs. HCV infection also causes endoplasmic reticulum (ER) stress. Further, HCV RNA replication was detected in MIN6 cells, although the infection efficiency is very low and no progeny virus particle generates. Taken together, our data suggest that HCV infection induces death of pancreatic beta cells through an ER stress-involved, caspase 3-dependent, special pathway.

## Introduction

Hepatitis C virus (HCV) has been recognized as a major cause of liver diseases and affects approximately 130–180 million people worldwide at the present time [1], [2]. Chronic infection with HCV induces chronic hepatitis, hepatic steatosis, cirrhosis and hepatocellular carcinoma [3], [4]. In addition to liver injury, there are multiple examples of extrahepatic disease attributed to HCV infection, such as mixed cryoglobulinemia, lichen planus, arthritis and other immunological disorders [5], [6]. Diabetes mellitus (DM), mostly type 2 DM (T2DM), is also an extrahepatic manifestation of HCV infection. Experimental and clinical studies have revealed that HCV infection is involved in the development of T2DM, and T2DM prevalence in HCV infection patients is much higher than that observed in the general population and in patients with other chronic liver diseases such as hepatitis B virus, alcoholic liver disease and cirrhosis [7], [8], [9], [10]. There is growing evidence to support the concept that HCV infection is a risk factor for developing T2DM. Evidence linking HCV infection and T2DM has mainly been obtained from retrospective case-control studies and/or studies performed in hospital-based settings. The biological mechanism underlying T2DM in HCV infection remains unknown. Recently, several clinical and experimental studies have supported the hypothesis that HCV may induce insulin resistance by interfering with insulin signaling [11], [12].

T2DM is a common endocrine disorder encompassing multifactorial pathogenetic mechanisms [13]. Although numerous studies have shown that insulin resistance precedes the development of hyperglycemia in patients that eventually develop T2DM [14], it is being recognized that T2DM only develops in insulin-resistant people displaying the onset of beta cell dysfunction [15], [16], [17], [18]. Multiple defects in insulin secretion and beta cell mass have been noted in patients with T2DM and also during the insulin-resistant prediabetic stage [19]. The concept of insufficient beta cell mass as the key factor in the pathogenesis of T2DM has recently been widely acknowledged. Beta cell mass plays an essential role in determining the amount of insulin that is secreted to maintain the body’s glucose levels within a narrow range [20], [21].

While HCV is known to replicate in the hepatocyte, the genome has been also identified in a number of other tissues including pancreas [22], [23], [24]. Acute insulin responsiveness is subnormal in patients with HCV infection, demonstrating that it is unlikely that insulin resistance alone causes diabetes without underlying impairment of beta cells [25]. Therefore, in addition to insulin resistance, patients with HCV infection may also have beta cell failure. However, potential effects of the virus on beta cells are not known and there is no in vitro model available to test the hypothesis that HCV directly damages human beta islet cells.

In the present study, by using the HCV infection system, we investigate the possible effect of HCV infection on the fate of MIN6 cells, a mouse insulin-producing pancreatic beta cell line which has been widely used for diabetes research. The data demonstrate that HCV represents an independent risk factor for physiologic control of beta cell death in the pathogenesis of diabetes development. Our study provides a rationale to investigate hepatitis C itself as a potential therapeutic target for treatment of HCV-associated T2DM.

## Results

### HCV Infection Decreases Cell Viability Directly in Insulinoma Cell Line

**Figure 1:**
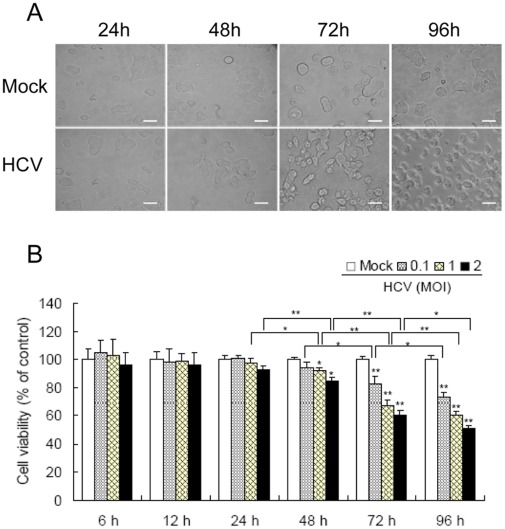
HCV infection decreases MIN6 cell viability. (**A**) The images of MIN6 cells infected with HCV at 24, 48, 72 and 96 hpi. MIN6 cells were mock-infected or infected with 1.0 MOI of the supernatant of HCV-infected Huh7.5.1. Scale bar, 10 µm. (**B**) MIN6 cells were infected with HCV at different MOIs. At indicated time points post-infection, the percentage of viable cells was assessed by MTT assay and plotted versus time. The activity measurements were done in triplicates. Data represent means + SD of three independent experiments (n = 9). *P<0.05, **P<0.01, compared with respective controls.

[]LOOSERMIN6 cells were incubated with the supernatants of HCV-infected Huh7.5.1 cells at 1.0 multiplicity of infection (MOI). In 24 hours post-infection (hpi), MIN6 cells morphologically resembled the three-dimensional islet-like structures of the mock-infected cells. From 48 hpi, MIN6 cells started to lose the homotypic beta-cell-to-beta-cell interactions. Then, the cells were singly dispersed instead of displaying individual cell-matrix interactions and underwent marked rounding, and eventually detached from the culture dish after incubation with HCV for 96 h (Fig. 1A), suggesting cell injury occurs at the late stage of virus infection.

Cell viability was then analyzed by the 3-(4,5-dimethylthiazol-2-yl)-2,5- diphenyltetrazo- lium bromide (MTT) method. At the early stage of HCV infection (6, 12 and 24 hpi), MIN6 cells exhibited no clear cytopathogenic effects, even at 2.0 MOI. At 48 hpi, HCV infection impaired cell viability in a dose-dependent manner. The threshold dose is 1.0 MOI (92.3+1.56% of control, *P*<0.05). Similar results were obtained at 72 and 96 hpi with the threshold dose of 0.1 MOI (Fig. 1B). Taken together, we demonstrated that HCV had a dose- and time-dependent cytopathogenic effect on MIN6 cell viability, evident at late stages of infection with high MOI.

**Figure 2:**
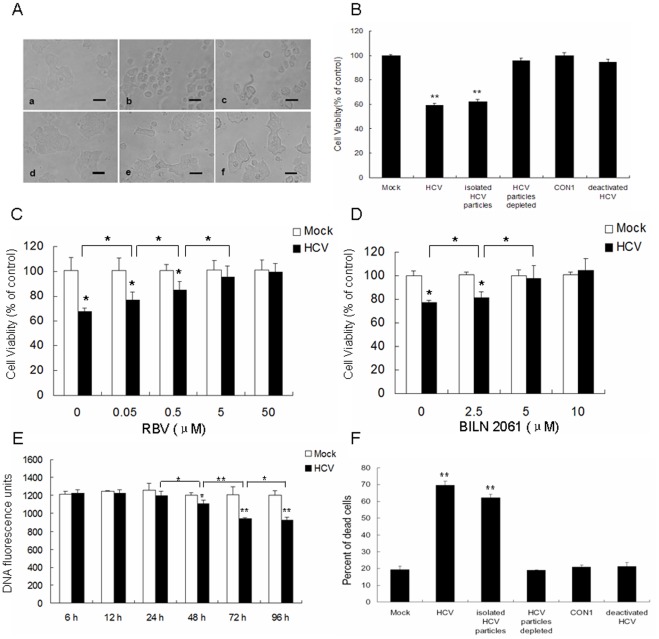
HCV played a direct role in the reduction of cell viability and induction of cell death of beta cells. (**A, B**) MIN6 cells were mock-infected (a) or infected with 1.0 MOI of the supernatant of HCV-infected Huh7.5.1 (b), ultracentrifugation-purified HCV particles (1.0 MOI) (c), the supernatant of HCV-infected Huh7.5.1 after ultracentrifugation (d), CON1 cell culture medium (e), or the supernatant of HCV-infected Huh7.5.1 after UV radiation treatment (f) at 96 hpi. Light microscopy images (scale bar, 10 µm) (**A**) were obtained and cell viability (**B**) of MIN6 cells was determined by MTT assay. (**C, D**) MIN6 cells were mock-infected or infected with HCV particles (1.0 MOI) with addition of indicated dose of RBV (**C**) or BILN 2061 (**D**) at 96 hpi, cell viability was assessed by MTT assay. (**E**) MIN6 cells were infected with HCV as in Figure 1B. At different time-points, cell number was determined by DNA content staining with the fluorescent dye PI. (**F**) MIN6 cells were treated as in (A). Cell death induced by the indicated treatment was measured by trypan blue exclusion. (**A–F**) All activity measurements were done in triplicates. Data represent means + SD of three independent experiments (n = 9). *P<0.05, **P<0.01, compared with respective controls.

To exclude the effects of proinflammatory cytokines from the viral culture supernatants, we analyzed the direct effect of HCV virion. The purified HCV particles induced MIN6 morphologic changes and decreased the number of metabolically active MIN6 cells (Fig. 2Ac and B), whereas virion-depleted culture supernatant had no effect (Fig. 2Ad and B), demonstrating that the virus particles were account for the impairment of cell viability. This was further confirmed by CON1 cell (with HCV subgenomic replicon) culture medium and UV-inactivated HCV supernatants (Fig. 2Ae, f and B). Furthermore, the addition of ribavirin (RBV) and a HCV-specific inhibitor, BILN 2061 could rescue the purified HCV particles induced-decreased cell viability of MIN6 cells in a dose-dependent manner (Fig. 2C and D). RBV (50 µM) and BILN 2061 (5 µM) completely inhibited the cytopathogenic effect of HCV on MIN6 cells. These results supported the hypothesis that HCV played a direct role in the reduction of cell viability of insulinoma beta cell.

### HCV Infection Induces MIN6 Cell Death Directly

**Figure 3:**
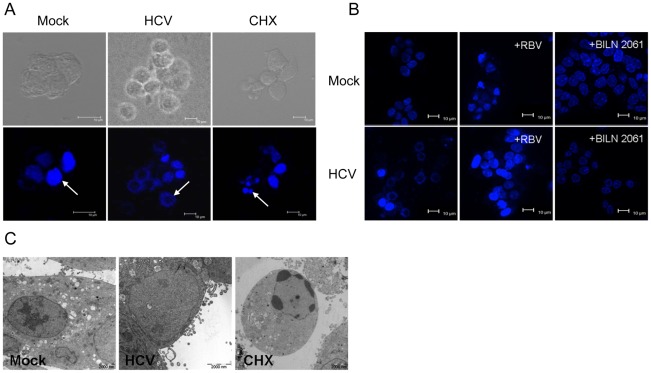
HCV infection induces novel morphological changes of MIN6 cells. (**A–C**) Confocal image of MIN6 cells stained with Hoechst 33258. Scale bar, 10 µm. Cells were mock infected or infected with 1.0 MOI HCV for 96 h. Cells treated with CHX for 48 h served as an apoptosis positive control. White arrows indicate nuclei (**A**). MIN6 cells were treated as in Figure 2A (**B**). Cells were mock-infected or infected with HCV particles (1.0 MOI) with addition of RBV (50 µM) or BILN 2061 (10 µM) at 96 hpi (**C**). (**D**) Electron microscopic analysis as in (A). Scale bar, 2 µm. All measurements were done in triplicates.

Maintenance of beta cell mass is critical for secretion of adequate amounts of insulin [20], [21]. We next examined the effects of HCV infection on pancreatic beta cell growth. Determination of DNA content by addition and measurement of the fluorescent dye propidium iodide (PI) indicated that the number of MIN6 cells was reduced dose-dependently from 48 hpi (Fig. 2E). Trypan blue exclusion cell counts were used to determine the death-inducing effect of HCV on MIN6 cells. As shown in Fig. 3B, a significant increase in the extent of cell death was observed in the HCV-incubated cells as compared to mock cells (69.6+2.43% vs 19.8+2.08%, *P*<0.01) at 96 hpi. As expected, the presence of isolated virus particles proved to be necessary to induce cell death (62.1+2.01%) and the virus-free culture supernatants resulted in no beta cell failure (18.9+0.28%). Similar results were obtained with CON1 culture medium and deactivated HCV (Fig. 2F). These data demonstrated that HCV inhibited pancreatic beta cell proliferation by inducing the death of MIN6 cells directly.

### HCV Induces Novel Morphological Changes of MIN6 Cells

Apoptosis reportedly plays a critical role in the reduction of beta cell mass occurring in patients with T2DM [26], [27]. HCV infected-MIN6 cells had remarkable morphological changes, whereas no apopotic body was detected (Fig. 1A). It is notable that the nucleus was enlarged and the chromatin staining with Hoechst circularized gradually after 48 h-HCV infection, which was different from the typical apoptosis with condensed chromatin as observed in cyclohexamide (CHX)-treated cells (Fig. 3A). Similar morphological changes were observed in isolated HCV particle-treated cells (Fig. S1a). Moreover, the viral particle-depleted supernatants, CON1 culture medium and deactivated HCV had no significant effect on the nucleus of MIN6 cells (Fig. S1). Inhibition of HCV replication in MIN6 cells with RBV or BILN 2061 also diminished the nucleus changes (Fig. 3B).

Further ultra-structural analyses revealed a dilated and low-density nucleus with the remaining plasma membrane in about 67% HCV-treated MIN6 cells, which was different with that observed in CHX-treated cells, which display nuclear fragmentation, typical chromatin condensation with marginalization at the nuclear periphery (Fig. 3C). These results indicated that HCV induced novel morphological changes of MIN6 cells.

### HCV Induces Apoptosis-like Death of MIN6 Cells

**Figure 4:**
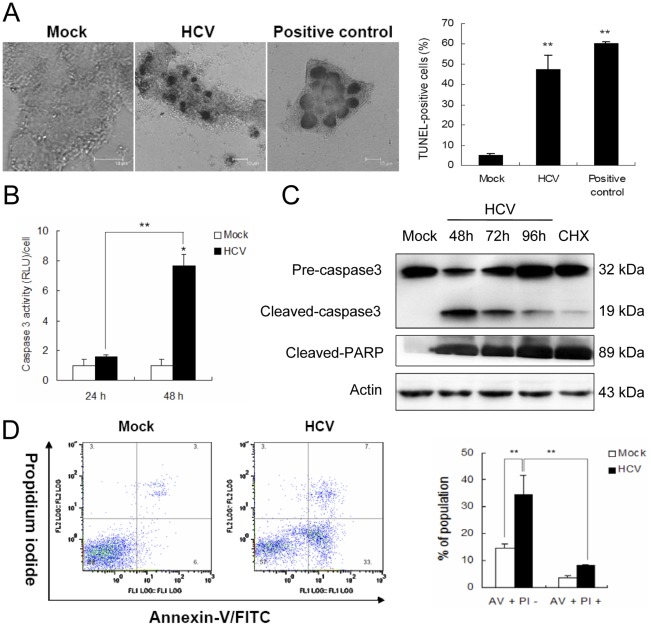
HCV infection induces apoptosis-like cell death in MIN6 cells. MIN6 cells were mock-infected or infected with 1.0 MOI of HCV. (**A**) Confocal image of cells stained with TUNEL at 96 hpi. Scale bar, 10 µm. Quantitative summary of the TUNEL-positive staining is provided on the left. (**B**) Caspase 3 activity levels at 24 and 48 hpi. The caspase 3 activity of the control cells at 0 h after treatment was arbitrarily expressed as 1.0. (**C**) Immunoblot analysis of caspase 3 and PARP at 48, 72, 96 hpi. Cells treated with CHX (50 ng/ml) for 48 h were served as an apoptosis positive control. Immunoblots are representative of at least three independent experiments. Amounts of actin were measured as an internal control to verify equivalent sample loading. (**D**) Diagrams of FITC-Annexin V/PI flow cytometry in a representative experiment at 48 hpi. Numbers in the quadrants indicate the proportions of cells in the corresponding areas (left panel). (**A, B, D**) All measurements were done in triplicates. Data represent means + SD of three independent experiments (n = 9). **P<0.01, compared with respective controls.

To further characterize HCV-induced cell death, transferase-mediated dUTP nick-end labeling (TUNEL) assay was performed. There were significantly fewer TUNEL-stained nuclei in the control groups, as compared to the groups incubated with 1.0 MOI HCV for 96 h, where nuclei fluoresced brightly, indicating fragmentation of DNA (Fig. 4A). The percentage of TUNEL-positive cells was significantly higher in HCV-infected MIN6 cells as compared to mock-infected cells (47.1+7.5% vs. 5.1+1.0%, *P*<0.01). It is important to note that, when the nuclei were stained with TUNEL, cells had intact cytoplasm and nuclear membranes.

Caspase enzymatic assays showed that caspase 3 activity in lysates from HCV-infected cells at 48 hpi was nearly four times higher than that in mock cell lysates, whereas DEVD cleavage activity was at background levels at 24 hpi (Fig. 4B). These results were confirmed by immunoblot analysis. Activated caspase 3 (19 kDa band) was detected in both HCV-infected MIN6 cells and CHX-treated positive control, but not in mock control (Fig. 4C). Analysis of the death substrate poly (ADP-ribose) polymerase (PARP) also demonstrated that PARP was cleaved to generate an 89-kDa fragment in HCV-infected cells (Fig. 4C), indicating that the caspase cascade is activated during HCV-induced cell death. Consistently, the proportion of annexin-V positive/PI-negative cells was evidently increased in HCV-incubated cultured cells, from 14.6+1.7% to 34+7.1%, implying the apoptotic nature of HCV-induced beta cell death (Fig. 4D).

**Figure 5:**
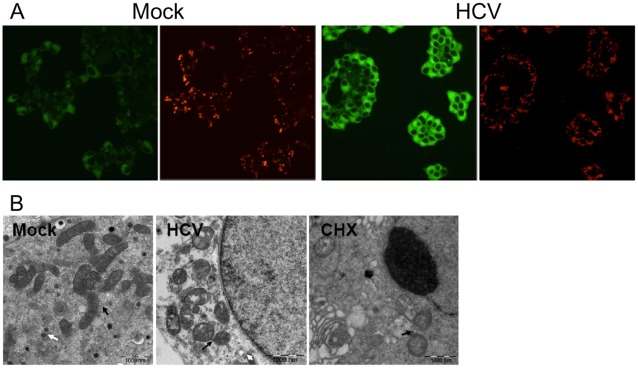
Mitochondrial changes in HCV-infected MIN6 cells. MIN6 cells were mock-infected or infected with 1.0 MOI of HCV. (**A**) Mitochondrial transmembrane potential changes at 48 hpi. Representative images from JC-1 staining are shown. The aggregated form of JC-1, characteristic of mitochondrial integrity (red) and monomeric JC-1 (green) were examined under laser confocal scanning microscope. (**B**) Electron microscopy of mitochondrial morphologies at 96 hpi. Cells treated with CHX (50 ng/ml) for 48 h were served as an apoptosis positive control. Black and white arrows indicate mitochondria and insulin granules, respectively. Scale bar, 1 µm.

Mitochondria play a central role in the regulation of both apoptotic and nonapoptotic cell death [28]. HCV-infected MIN6 cells stained with the vital mitochondrial dye JC-1, showing the elimination of red J-aggregate fluorescence and cytoplasmic diffusion of green monomer fluorescence as compared to the mock one, suggesting a loss of mitochondrial membrane potential (Fig. 5A). We further detected the loss of Rhodamine 123 (Rho123) fluorescence intensity, which was similar to the result in CHX-induced apoptosis (9.7+0.37% vs. 11.6+0.5%), revealing that HCV influenced the mitochondrial transmembrane potential in MIN6 cells (Fig. S2). Transmission electron microscopy also exhibited swelling, cristae vague and structural alterations of mitochondria in HCV-infected MIN6 cells (Fig. 5B), suggesting that mitochondria was involved in the regulation of this special cell death. Based on the above observations, we proposed that HCV-infected cells died through an apoptosis-like mechanism, highlighting a novel functionality for HCV that is distinct from its role in infecting hepatocytes and inducing apoptosis.

### HCV Induces ER Stress in MIN6 Cells

**Figure 6:**
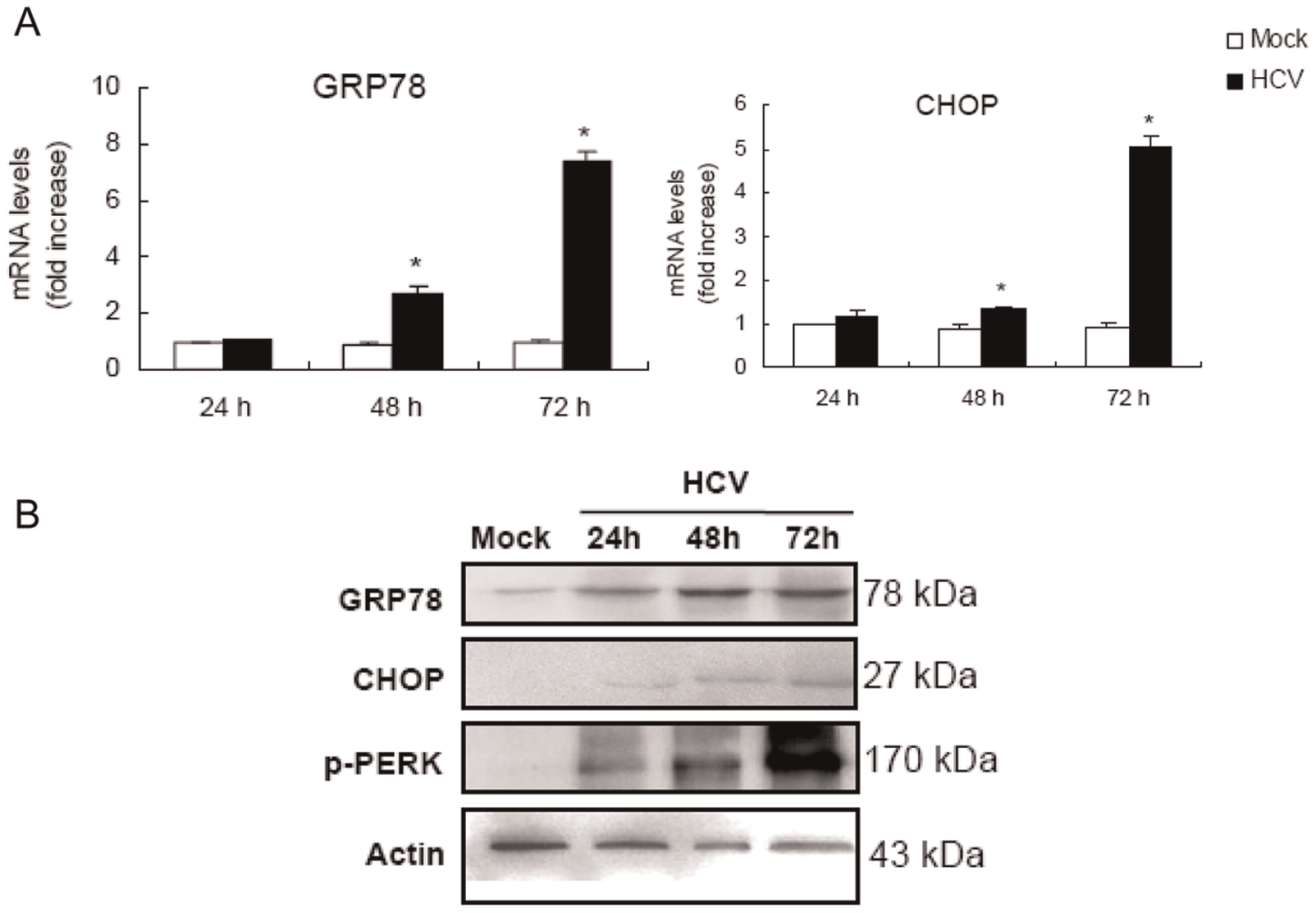
HCV infection induces ER stress in MIN6 cells. (**A**) mRNA levels of GRP78 and CHOP. MIN6 cells were mock-infected or infected with 1.0 MOI HCV for 24, 48, 72 h. mRNA was analyzed by real-time RT-PCR. The results were normalized with the values obtained from actin in the same sample. Data are expressed as fold-increase relative to the values observed in mock control. The measurements were done in triplicates. Data represent means + SD of three independent experiments (n = 9). *P<0.05, compared with respective controls. (**B**) Immunoblot analysis of GRP78, CHOP and p-PERK corresponding to (A) probed with the indicated antibodies. Amounts of actin were measured as an internal control to verify equivalent sample loading. Immunoblots are representative of at least three independent experiments.

Beta cells are very susceptible to endoplasmic reticulum (ER) stress and chronic ER stress during the development of diabetes could potentially lead to beta cells loss [29]. Therefore, we examined whether HCV infection induced ER stress in MIN6 cells. The 78-kDa glucose-regulated protein (GRP78) is an ER chaperone of which expression is increased upon ER stress. Both the mRNA and protein levels of GRP78 were found to be significantly elevated from 24 to 72 hpi specifically in HCV infected-MIN6 cells as compared to the control (Fig. 6). The expression patterns of another ER stress marker, PKR-like kinase (PERK), were also examined and exhibited an increased phosphorylation status in HCV-infected MIN6 cells (Fig. 6B). These data strongly suggested that the ER stress is an early cell response of MIN6 cells challenged with HCV. HCV infection also mediated an increased expression of C/EBP homologous protein (CHOP) (Fig. 6), which plays an important role as ER stress-induced cell death modulator. Taken together, these results demonstrated that ER stress might play an important role in HCV-induced apoptosis-like death of MIN6 cells.

### HCV Replicates in MIN6 Cells

**Figure 7:**
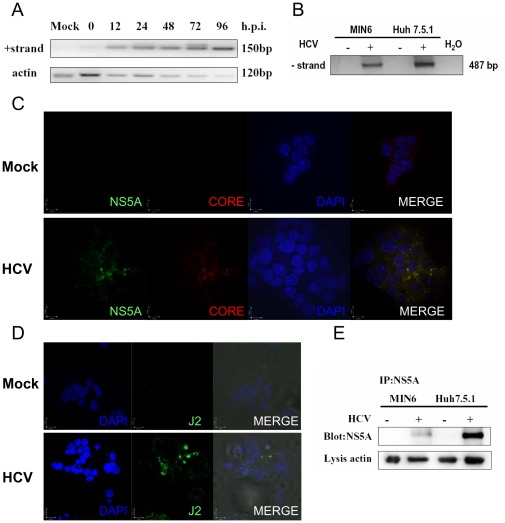
Hepatitis C virus replicates in MIN6 cells. (**A**) Kinetics of detection of positive-strand HCV RNA. MIN6 and Huh7.5.1 cells were mock-infected or infected with 1.0 MOI HCV. At different time points, cells were harvested and total RNA was isolated and reverse-transcribed to cDNA. Single-round PCR products (150 bp) were obtained by amplification with POSF/R primers. Actin was measured as an internal control. (**B**) Detection of the synthetic negative-strand RNA as in (A). The sample from HCV-infected Huh7.5.1 cells was used as the positive control. Negative controls included PCR amplification from non-infected cells (−) and water (H_2_O). (**C–D**) MIN6 cells were mock-infected or infected with 1.0 MOI of HCV at 24 hpi. HCV core (red) and NS5A (green) labeled with respective antibody (**C**) or HCV dsRNA labeled with J2 antibody (green) (**D**) were examined by immunofluorescence assay. Blue fluorescence represents DAPI-stained nuclei as observed. (**E**) Immunoprecipitation and blotting of NS5A purified from 96 h-infected MIN6 and Huh7.5.1 cells. Actin from lysis was used as the internal control. All measurements were done in triplicates. Immunoblots are representative of at least three independent experiments.

It has been reported that HCV is not strictly hepatotropic and the common presence of negative-strand HCV RNA is detected in pancreas from HCV-infected patients with AIDS [22]. As shown in Fig. 7A, starting from 12 hpi, positive-strand HCV RNA was already detectable in the HCV-infected MIN6 cells and increased with prolongation of infection time. Quantitative RT-PCR analysis confirmed the increase of positive-strand RNA as well (data not shown). The presence of HCV negative strand in infected MIN6 cells was also revealed by using a negative-strand-specific nested-PCR assay (Fig. 7B). Immunostaining for both core and NS5A protein (Fig. 7C) and further HCV dsRNA (Fig. 7D) demonstrated an infection of HCV in MIN6 cells by immunofluorescence assay. Moreover the translation products NS5A in MIN6 cells were probed with anti-NS5A after enrichment by using immunoprecipitation (Fig. 7E). These results revealed that HCV could replicate in the insulinoma MIN6 cells with low efficiency, although the detailed HCV-entry mechanism of MIN6 cells remained obscure.

## Discussion

Since the discovery of HCV in 1989, emerging evidence has shown that the chronic HCV infection is associated with an increased risk of developing T2DM [30]. In addition to insulin resistance, the pancreatic beta cell dysfunction is central to the progression of T2DM. The mechanism of beta cell destruction, either directly by HCV or by an immune response, remains unclear. In the present study, we investigate the HCV-induced cytotoxicity and its cellular mechanism in pancreatic beta cells by using a virus infection system. The results show that HCV possessed the capacity to induce beta cell death directly.

HCV only infects humans and chimpanzees, indicating that specific host factors are required for HCV entry, RNA replication, assembly, and release of virions. However, HCV RNA replication has been observed in non-hepatic human cells and even nonhuman cells, although the replication efficiency is rather low [31], [32]. Here, we provide evidence indicating the low level of replication and translation of the HCV genome in MIN6 cells. Nevertheless, we could not detect the release of progeny virus particles in HCV-infected beta cells (data not shown). This result is consistent with previous observations that mouse cells have been shown to sustain HCV RNA replication, although the efficiency is low and release of progeny virus particles is not observed [32]. These findings raise the possibility that HCV may employ different mechanisms, such as other receptor molecules that mediate the entry and replication of HCV in the extrahepatic tissue, such as pancreatic cells and even nonhuman cells.

There is increasing evidence that the inflammatory process plays a pivotal role in islets of patients with T2DM [33]. HCV infection promotes insulin resistance, mainly through increased tumor necrosis factor α (TNF-α), which inhibit insulin receptor and IRS-1 (insulin receptor substrate) tyrosine phosphorylation [34], [35]. It has been reported that the elevated inflammatory cytokines, especially TNF-α in the liver and serum in chronic hepatitis C, suggesting a possible link between HCV infection, inflammatory cytokines, and insulin resistance [36]. However, TNF-α induction in liver disease is by no means unique to chronic hepatitis C. Other types of liver infection and inflammation such as chronic hepatitis B and alcoholic hepatitis, which have no significant prevalence with T2DM, also induce TNF-α [37], [38]. The fact that the viral particle-depleted supernatants and the CON1 culture medium have no significant effect on MIN6 cells indicates that cellular factors produced in HCV infected-hepatocyted do not affect pancreatic beta cell. Our data also strongly indicates that HCV infection is essential for the cell death of pancreatic cells. These results may partly explain the observed predilection of the development of DM in non-cirrhotic HCV-infected patients and an indispensable role of HCV in these processes.

Viruses have been implicated as one environmental factor that may initiate or trigger an autoimmune reaction that targets and destroys beta cells in genetically susceptible individuals [39]. However, diabetes in HCV-positive patients is not specifically associated with autoantibody production, so autoimmune destruction of the pancreas does not seem to be an important mechanism [40]. Accordingly, HCV infection may directly damage beta cells and disturb their function, resulting in T2DM. Consistent with our results, the HCV genome has been identified in pancreatic cells [23]. Furthermore, a direct cytopathogenic effect of HCV at the islet cell level has been documented [24]. In addition, our data supports previous studies from an epidemiological setting, which reported that HCV viremia, but not anti-HCV seropositivity alone, associates with T2DM. These results imply that a persistent and/or active phase of HCV infection is associated with T2DM [9].

Beta cells normally undergo compensatory changes to meet the metabolic demand, and succumb to the pressure that leads to a subsequent reduction in beta cell mass, which eventually develops to T2DM [41]. Although beta cell mass is controlled by beta cell replication, size, neogenesis, and death, studies have shown that the loss of beta cell mass is primarily due to a reduced number of beta cell, as a result of increasing cell death [26]. In this study, we show that significant reduction in proliferation of MIN6 cells was found and associated with the beta cell death induced by HCV at the late stage of infection. To our surprise, the percent of cell death disagrees with the low efficiency of replication. One reason might be due to the feature of the MIN6 cell line which morphologically resemble as the three-dimensional islet-like structures with cell-to-cell interactions. It has been reported that the cell-to-cell contact increases the rate of apoptosis [42]. We are keen to explore the underlying mechanisms further.

Many findings indicate that in T2DM, a relative reduction of beta cell mass occurs as the result of excessive apoptosis [43]. However, HCV-infected MIN6 cells displayed a dilated and low-density nucleus, differed from the typical apoptosis. Although our observation does not completely coincide with the other view that apoptosis is major type of beta cell death in diabetes [44], our results are consistent with the study showing that the absence of increased apoptosis was observed in diabetic patients with HCV infection [24].

The mechanism of HCV-mediated cell death remains unclear. However, the observation that HCV increased activated caspase 3 as well as other typical apoptotic characteristics (positive TUNEL etc.) points to an apoptosis-like feature of beta cells. Of note, as shown in Fig. 4C, the expression patterns of cleaved caspase 3 have exhibited a special decrease status from 48 to 96 hpi, which might give a clue to further investigate the molecular mechanism of HCV-induced beta cell death.

An increasing amount of evidence shows the link between ER stress and beta cell failure in T2DM. Beta cell death in the Akita diabetic [29] and NOD.k iHEL nonimmune [45] diabetic mouse models is attributed to ER stress. Studies have shown that mice lacking PERK were found to develop hyperglycemia and increased beta cell death [46]. In addition, the disruption of CHOP delayed the onset of diabetes in the Akita mice [29]. The increased expressing of ER stress marker (GRP78, CHOP, and PERK) indicates that HCV-incubated beta cells response to ER stress and it is reasonable to assume that the ER stress-mediated pathway is crucially involved in HCV induced apoptosis-like death of beta cells. As the current understanding of events activated during ER stress-induced cellular events accumulates, it is recognized that prolonged ER stress can lead to mitochondrial damage [47]. Actually, HCV infection is correlated with the loss of mitochondrial membrane potential as well as the damage of mitochondria in MIN6 cells. In agreement, we also found the activation of caspase 9 (Fig. S3), which in turn activates caspase 3, results in DNA fragmentation and cell death. These findings raise the possibility that HCV-induced beta cell death due to ER stress requires the activation of the mitochondria.

In summary, we employed a unique virus infection system to directly demonstrate the characteristics of HCV-infected beta cells. These findings suggest that HCV persistent infection may induce a special beta cell death through ER stress-involved, mitochondria activated, caspase 3-dependent pathway. It is tempting to speculate that HCV in pancreatic beta cells is one factor that contributes to beta cell failure and consequent glucose intolerance in HCV-associated DM patients.

## Materials and Methods

### Reagents

MTT, RBV, CHX, PI, Rho123, trypan blue and Hoechst 33258 were purchased from Sigma (St. Louis, USA). Antibodies against caspase3, cleaved-caspase3, PARP, GRP78, PERK phosphorylated at Thr^981^ (p-PERK), and CHOP were purchased from Cell Signaling Technology (Beverly, USA). Anti-NS5A and core antibody were kindly provided by Dr. C, Rice (Rockefeller University, USA). Anti-actin, insulin and horseradish peroxidase (HRP) - conjugated anti-rabbit or anti-goat immunoglobulin G (IgG) antibodies were purchased from Santa Cruz Biotechnology (CA, USA). Alexa 488-, 568- or 647-conjugated donkey anti-mouse, anti-rabbit or anti-goat IgG antibodies were obtained from Invitrogen (CA, USA). HCV NS3 protease inhibitor BILN 2061 (Boehringer Ingelheim, Germany) and dsRNA antibody J2 (Engscicons, Hungary) were used in the researches.

### Cell Culture

Human hepatoma cell lines Huh7.5.1 (kindly provided by Prof. F. V. Chisari, The Scripps Research Institute, USA), CON1 (Huh7-lunet with subgenomic HCV replicon) [48] and the insulin-secreting mouse cell line MIN6 [49] were cultured respectively as previously described.

### Virus Productions and Infections

Plasmid pJFH1 (kindly provided by Dr. Wakita, National Institute of Infectious Diseases, Tokyo, Japan) were used in this study. The viral stocks were produced by infection of naïve Huh7.5.1 cells with medium collected from Huh7.5.1 cells electroporated with in vitro transcribed pJFH1 RNA as previously described [50]. Virus purification was performed as previously described [32]. Virus infections were performed at a MOI of 1.0 or as indicated. Mock infections were performed in parallel to virus infections, but in the absence of virus.

### Cell Viability/Proliferation/Death Assays

Cell viability was analyzed by MTT methods according to the manufacturer’s instructions (Sigma). Briefly, MIN6 cells (1.6×10^4^ cells/well) were plated in 96-well platea and infected with various concentrations of HCV or the indicated treatments. Thereafter, cells were washed twice with phosphate-buffered saline (PBS) and incubated with 0.1 mg/ml MTT for 4 h at 37°C and 5% CO_2_. Formazan crystals were then dissolved in 150 µl of DMSO by incubation for 15 min at room temperature and the optical density was detected at 570 nm using a fluorescence microplate reader (BioTek Instruments). DNA fluorometric assay was used to assess the effects of HCV infection on the proliferation of pancreatic beta cells using the fluorescent dye PI as described previously [51]. Cell death was assessed by trypan blue exclusion according to the manufacturer’s instructions (Sigma).

### Fluorescence Microscopic Analysis

MIN6 cells (1×10^6^) grown on glass coverslips in petri dishes were rinsed twice, fixed in 4% paraformaldehyde for 10 min on ice, permeabilized with 0.15% Triton X-100, stained with Hoechst 33258 (1 µg/ml), and then examined under a Leica S2 laser confocal scanning microscope (Leica TCS NT, Heidelberg, Germany).

### Electron Microscopy Analysis

Samples for electron microscopy were prepared as described previously [52] and examined with the FEI Tecnai™ transmission electron microscope G2 20 Twin (Hillsboro, USA).

### TUNEL Assay

Cells were seeded, rinsed, fixed, permeabilized and submitted to the TUNEL assay using the DeadEnd™ Colorimetric Tunel Systerm (Promega, WI, USA) according to the manufacturer’s instructions. Results are expressed as percentage of TUNEL-positive cells.

### Caspase Enzymatic Activity

Caspase 3 activity was measured using the Caspase-Glo 3/7 assay kit (Promega) and a Luminometer 20/20 n (Turner BioSystems, Sunnyvale, CA, USA) according to the manufacturer’s instructions.

### Detection of Phosphatidylserine Externalization

HCV-infected MIN6 cells (1.5×10^6^) harvested at 48 hpi were subjected to measure the translocation of phosphatidylserine according to the manufacturer’s instructions (Keygene, China). Samples were immediately submitted to fluorescence-activated cell sorting (FACS) using a Beckman Coulter Epics XL Flow Cytometer (USA) excited by the 488 nm line of an argon-ion laser.

### Measurement of the Mitochondrial Transmembrane Potential

The fluorescent probe JC-1 (Molecular Probes, Eugene, OR, USA), a cationic membrane potential indicator, was used to assess the mitochondrial membrane potential according to the manufacturer’s protocol. MIN6 cells in 35 mm dishes were incubated with 2 µg/ml JC-1 in growth medium at 37°C for 30 min. The cultures were washed three times using fresh growth medium. Mitochondria were then analyzed immediately under a Leica S2 laser confocal scanning microscope with a dual emission fluorescence filter with 515–545 nm for green fluorescence and emission at 585–615 nm for red fluorescence.

### Real-time RT-PCR

Total RNA was extracted from cells by using Trizol reagent (Invitrogen) according to the supplier’s protocol, and 2.0 µg total RNA were reverse-transcribed into cDNA using M-MLV reverse transcriptase (Promega). Reverse transcription-polymerase chain reaction (RT-PCR) of HCV positive- and negative-strand RNA were performed as described [53] with gene-specific primers (GSPs) (Table S1). Transcription levels of indicated genes and actin were measured by Real-time RT-PCR in triplicate using SYBR PCR master mix (Toyobo, Japan) with indicated GSPs (Table S1), and the StepOne Real-Time PCR System (Applied Biosystems, CA, USA), according to the manufacturer’s instructions. The quantification values of each specific gene were normalized to actin, which served as an endogenous reference gene.

### Immunoblotting

Cells were incubated in radio-immuno precipitation assay (RIPA) buffer on ice for 15 min. Total protein (30 µg) were loaded onto 10–15% SDS-PAGE and immunoblotting was performed as described [54]. The immunoreactive bands were then detected using an enhanced chemiluminescence detection system (Amersham Pharmacia Biotech, NJ, USA). Protein loadings were normalized by probing with anti-actin.

### Immunoprecipitation

HCV-infected MIN6 cells were lysed with ice-cold RIPA buffer at 96 h post infection (hpi), 4 µg anti-NS5A antibody was added to the lysate containing 200 µg of total protein, followed by overnight incubation at 4°C with continuous rotation. 20 µl immobilized protein A beads (Invitrogen) were then added to the samples, which were incubated at 4°C for 4 h with continuous rotation. The eluates were then subjected to SDS-PAGE, followed by immunoblotting, as described above.

### Immunofluorescence

Indirect immunofluorescence was performed as described previously [55]. Cells grown on glass coverslips in petri dishes were rinsed twice, fixed in 4% paraformaldehyde for 10 min on ice, permeabilized with 0.15% Triton X-100. Then cells were incubated with the indicated primary antibodies and the fluorescent secondary antibodies. Nuclei were stained with Hoechst 33258. The stainings were examined under a TCS-SP2 laser confocal scanning microscope.

### Statistical Analysis

Data are expressed as means + SDs. The two-tailed Student’s t-test was applied to evaluate the statistical significance of differences measured from the data sets. A *P* value of <0.05 was considered statistically significant.

## Supporting Information

Figure S1 **Confocal image of MIN6 cells stained with Hoechst 33258.** Cells were mock infected (a) or infected with HCV particles (1.0 MOI) (b), the supernatant of HCV-infected Huh7.5.1 after ultracentrifugation (c), CON1 cell culture medium (d), or UV radiation treatment HCV (e) at 96 hpi. Scale bar, 10 µm.
(TIF)

Figure S2 **Mitochondrial transmembrane potential changes at 48 hpi.** MIN6 cells were mock-infected or infected with 1.0 MOI of HCV. Cells treated with CHX (50 ng/ml) for 48 h were served as an apoptosis positive control. The proportions of cells with reduced Rho123 staining are shown. Data are presented from one experiment. Data represent means + SD of three independent experiments (n = 9).
(TIF)

Figure S3 **Immunoblot analysis of caspase 9 at 48, 72, 96 hpi.** MIN6 cells treated with CHX (50 ng/ml) for 48 h were served as an apoptosis positive control. Amounts of actin were measured as an internal control to verify equivalent sample loading. Immunoblots are representative of at least three independent experiments.
(TIF)

Table S1 **Primers used in this study.**
(DOCX)
