# Supplementary figures and images for: Hepatitis C Virus Induced a Novel Apoptosis-Like Death of Pancreatic Beta Cells through a Caspase 3-Dependent Pathway
Source: PLoS One. 2012 Jun 4;7(6):e38522. doi: 10.1371/journal.pone.0038522 (PMC3366942; doi:10.1371/journal.pone.0038522)

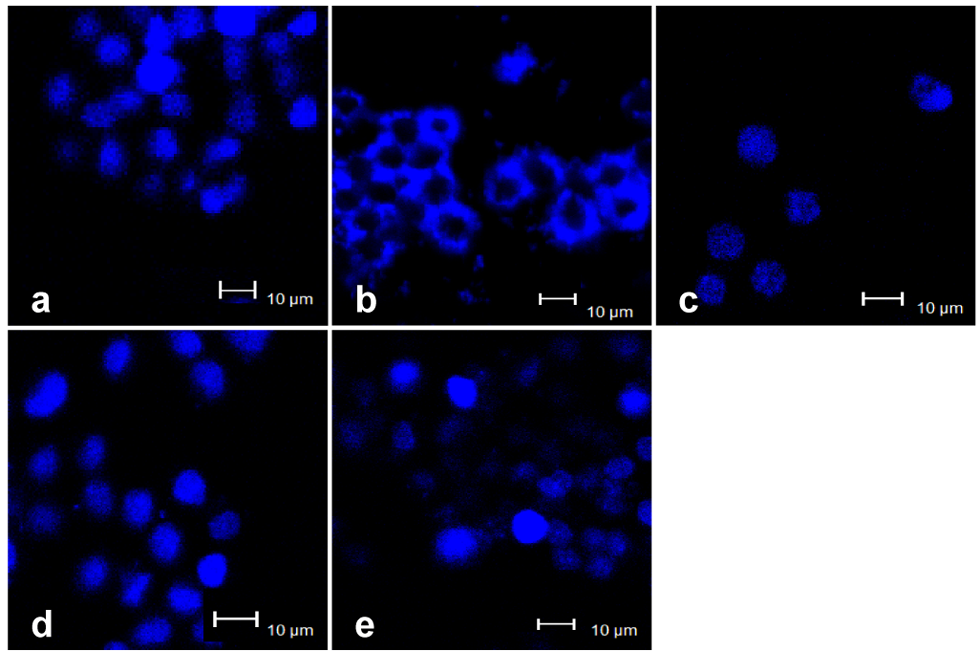

Supplement: Figure S1 — Confocal image of MIN6 cells stained with Hoechst 33258. Cells were mock infected (a) or infected with HCV particles (1.0 MOI) (b), the supernatant of HCV-infected Huh7.5.1 after ultracentrifugation (c), CON1 cell culture medium (d), or UV radiation treatment HCV (e) at 96 hpi. Scale bar, 10 µm. (TIF) [file pone.0038522.s001.tif]

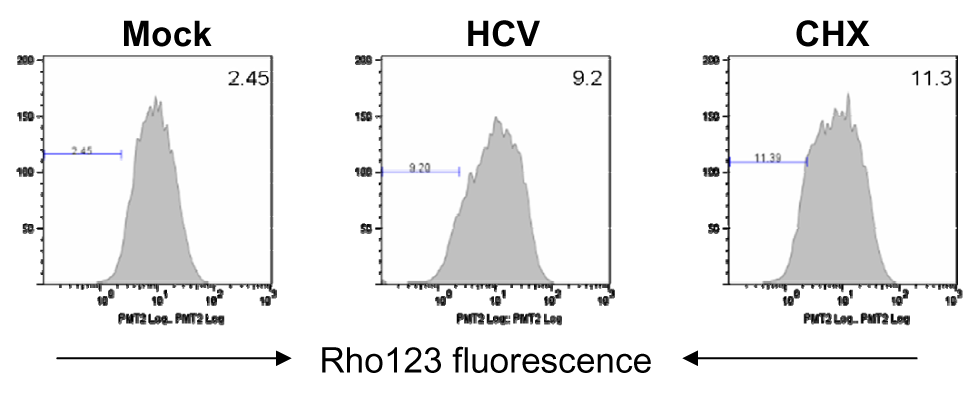

Supplement: Figure S2 — Mitochondrial transmembrane potential changes at 48 hpi. MIN6 cells were mock-infected or infected with 1.0 MOI of HCV. Cells treated with CHX (50 ng/ml) for 48 h were served as an apoptosis positive control. The proportions of cells with reduced Rho123 staining are shown. Data are presented from one experiment. Data represent means + SD of three independent experiments (n = 9). (TIF) [file pone.0038522.s002.tif]

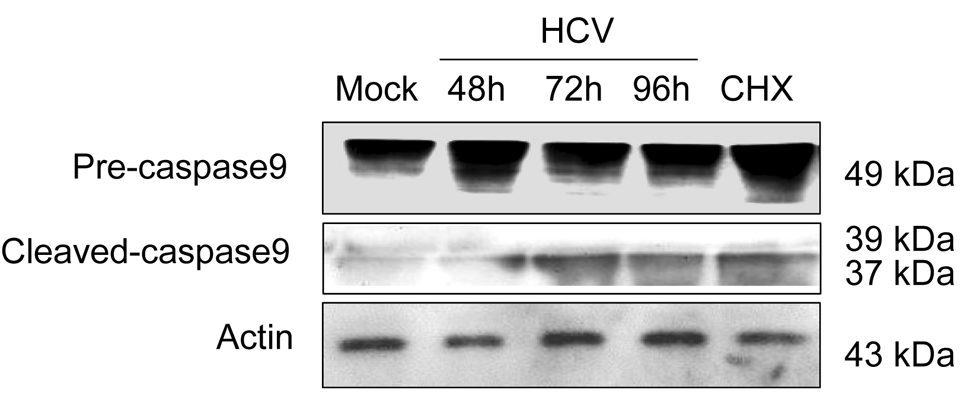

Supplement: Figure S3 — Immunoblot analysis of caspase 9 at 48, 72, 96 hpi. MIN6 cells treated with CHX (50 ng/ml) for 48 h were served as an apoptosis positive control. Amounts of actin were measured as an internal control to verify equivalent sample loading. Immunoblots are representative of at least three independent experiments. (TIF) [file pone.0038522.s003.tif]
