# Supplementary material for: Hepatitis C Virus Induced a Novel Apoptosis-Like Death of Pancreatic Beta Cells through a Caspase 3-Dependent Pathway
Source: PLoS One. 2012 Jun 4;7(6):e38522. doi: 10.1371/journal.pone.0038522 (PMC3366942; doi:10.1371/journal.pone.0038522)
Supplement: Table S1 — Primers used in this study. (DOCX) [file pone.0038522.s004.docx]

**Table S1. Primers used in this study**

| Strand/Gene | Primer name | Sequence (5’-3’) |
| --- | --- | --- |
| HCV Positive-strand | POSF POSL | TCTGCGGAACCGGTGAGTA TCAGGCAGTACCACAAGGC |
| HCV Negative-strand | NEGF NEGL | TGTCATGGTGGCGAATAA  CGCGGCAAGTAAA |
| Actin | ActinF ActinR | AGGCCAACCGTGAAAAGATC AGAGCATAGCCCTCGTAGATGG |
| GRP78 | GRP78F GRP78R | CTGGGTACATTTGATCTGACTGG GCATCCTGGTGGCTTTCCAGCCATTC |
| CHOP | CHOPF CHOPR | GAAGCCTGGTATGAGGATCT TCTGACTGGAATCTGGAGAG |
